# Codon-level co-occurrences of germline variants and somatic mutations in cancer are rare but often lead to incorrect variant annotation and underestimated impact prediction

**DOI:** 10.1371/journal.pone.0174766

**Published:** 2017-03-28

**Authors:** Amanda Koire, Young Won Kim, Jarey Wang, Panagiotis Katsonis, Haijing Jin, Olivier Lichtarge

**Affiliations:** 1 Program in Structural and Computational Biology and Molecular Biophysics, Baylor College of Medicine, Houston, Texas, United States of America; 2 Medical Scientist Training Program, Baylor College of Medicine, Houston, Texas, United States of America; 3 Program in Integrative Molecular and Biomedical Sciences, Baylor College of Medicine, Houston, Texas, United States of America; 4 Program in Translational Biology and Molecular Medicine, Baylor College of Medicine, Houston, Texas, United States of America; 5 Department of Molecular and Human Genetics, Baylor College of Medicine, Houston, Texas, United States of America; CNR, ITALY

## Abstract

Cancer cells explore a broad mutational landscape, bringing the possibility that tumor-specific somatic mutations could fall in the same codons as germline SNVs and leverage their presence to produce substitutions with a larger impact on protein function. While multiple, temporally consecutive mutations to the same codon have in the past been detected in the germline, this phenomenon has not yet been explored in the context of germline-somatic variant co-occurrences during cancer development. We examined germline context at somatic mutation sites for 1395 patients across four cancer cohorts (breast, skin, colon, and head and neck) and found 392 codon-level co-occurrences between germline and somatic variants, including over a dozen in well-known cancer genes. We found that for the majority of these co-occurrence events, traditional somatic calling led to an inaccurate representation of the protein site and a significantly lower predicted impact on protein fitness. We conclude that these events often lead to imprecise annotation of somatic variants but do not appear to be a frequent source of driver events during cancer development.

## Introduction

There is substantial genomic variation between individuals in the human population, with more than four million DNA differences between two random people [1], the overwhelming majority of which are single nucleotide variations (SNVs) [2]. SNVs are present even in the coding regions of genes related to cancer—amongst germline mutations gathered from the first phase of the 1000 Genomes Project [1], variants can be detected in more than eighty percent of COSMIC census cancer genes [3]. How these variants contribute on their own to cancer risk and development has long been a topic of interest, from family-based studies to identify rare variants that dramatically increase cancer risk [4–5] to GWAS to identify common SNPs that may modulate disease [6–7]. However, less is known about how inherited germline variants interact with the somatic variants gained during tumor development. Most recently, studies have approached this question by focusing on epistasis between inherited and tumor-specific variants that occur in different genes, and have identified pairs of genes that demonstrate significantly co-occurring or mutually exclusive germline and somatic mutations [8–10]. These findings suggest that pre-existing germline variants can interact indirectly with somatic variants to influence which somatic variants undergo positive selection.

However, germline and somatic variants may interact in a more direct fashion. As cancer cells evolve to explore a broad mutational landscape [11], tumor-specific somatic mutations may fall in the same codons as germline SNVs. The existence of cases in which multiple variants affect a single codon has been previously established by studies that identified dinucleotide polymorphisms (DNPs) in germline DNA of healthy individuals [12], and, separately, somatic variation across eight cancer types [13]. However, this past research only examined immediately adjacent base changes and did not respect codon boundaries or consider impact to amino acid predictions. Furthermore, because these studies considered germline and somatic variants independently, they considered only temporally ‘nonconsecutive’ cases where the two SNVs occurred at the same point in development. However, there are at least some described cases of DNPs produced in a temporally consecutive manner: a CC>TG mutation causing Alagille syndrome [14], as well as 15 other germline DNPs deemed ‘consecutive’ in HGMD [13]. Therefore, while past work supports the possibility that multiple, temporally consecutive mutations in the same codon can occur and be detected, this phenomenon does not appear to have been explored in the context of germline-somatic variant co-occurrences during cancer development.

**Fig 1:**
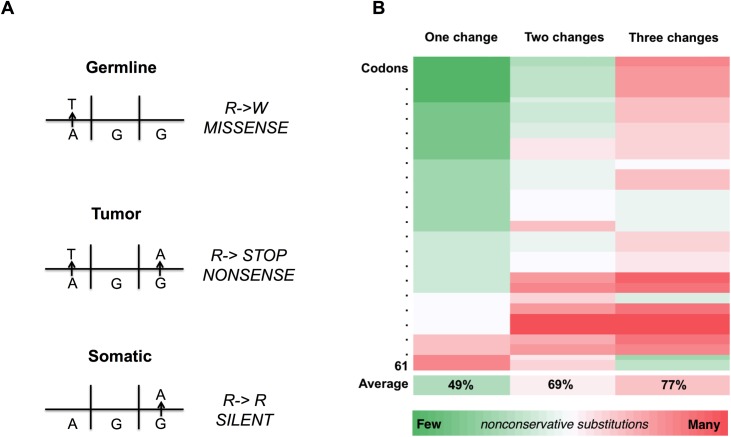
Co-occurrence of germline and somatic variants in a codon can lead to incorrect somatic calling and less conservative amino acid substitutions. (A) Overlooking germline context during somatic calling can lead to incorrect calls. (B) Relationship between the number of changes to the nucleotides in a codon and the proportion of resultant nonconservative amino acid substitutions. Each row represents one of the 61 codons capable of encoding an amino acid. Nonconservative substitutions were considered nonsense substitutions or missense substitutions that altered the classification of the substitution as polar (G, S, T, C, Y, N, Q), nonpolar (A, V, L, I, P, F, W, M), acidic (D, E) or basic (K, R, H). The final row indicates the average proportion of nonconservative amino acid substitutions resulting from one, two, or three nucleotide changes, across the 61 codons representing amino acids.

When these events occur, current methods for somatic variant calling ignore the germline context and can potentially annotate the resultant amino acid change incorrectly (Fig 1A). Since multiple changes to a codon are more likely to result in a stopgain or non-conservative amino acid change (Fig 1B), such incorrect annotations are likely to underestimate the true impact of the variants on protein fitness. In this way, tumor-specific variants could be leveraging existing germline variants to produce mutations with larger effects on phenotype. To assess the prevalence and impact of these events, and to identify whether these events undergo positive selection during tumorigenesis, we assessed the germline and somatic variant calls for four tumor types representative of different points along the somatic mutation rate spectrum: breast cancer (BRCA), head and neck squamous cell carcinoma (HNSC), colon adenocarcinoma (COAD), and skin cancer (SKCM). By identifying somatic variants within two nucleotides of a germline variant, and subsequently visualizing these sites in the tumor read alignment files, we detected hundreds of codon-level co-occurrences between germline and somatic variants. We found that while these events do not appear to be under positive selection during tumorigenesis, the somatic variant call was not an accurate representation of the protein site in the majority of cases where these events occurred. When the germline contexts of the somatic variants were considered, the amino acid changes and their predicted impacts were significantly larger. We conclude that these events often lead to imprecise annotation of somatic variants but do not appear to be a frequent source of driver events during cancer development.

## Materials and methods

### Data acquisition and quality assessment

Germline exome variant calls and somatic variant calls were downloaded on 10/23/14 from the Cancer Genomics Hub (CGHub), the central repository for source files from The Cancer Genome Atlas. For the germline exomes, the skin cancer (SKCM) and colon cancer (COAD) cohorts were sequenced on an Illumina GA DNA sequencing platform at Baylor College of Medicine, the breast cancer (BRCA) cohort was sequenced on an Illumina GA DNA sequencing platform at UC Santa Cruz, and the head and neck squamous cell carcinoma (HNSC) cohort was sequenced on an Illumina GA DNA sequencing platform at the Broad Institute. These cohorts were comprised of 343, 213, 501, and 338 patients respectively. The germline exomes displayed high quality, with an average TiTv value of 2.94±0.29, and a lambda value of 0.036±0.004 indicating that an average exome had fewer than 5% predicted false positive calls [15]. For somatic variants, we used COAD and SKCM somatic calls by Atlas2 at Baylor College of Medicine, BRCA somatic calls by bambam at UC Santa Cruz, and HNSC somatic calls by GATK at the Broad Institute. In all cases, only variants denoted as passing were considered for analysis. Tumor and germline variant allele fractions for patients were derived from the information associated with each reported call. Tumor read alignment files were downloaded from CGhub between 3/13/2016 and 7/7/2016.

Cancer genes were defined by the 595 genes in the Catalogue of Somatic Mutations in Cancer (COSMIC) cancer gene census [3]. The most current version of the cancer gene census was downloaded 7/22/16, and this version had last been updated by COSMIC on 3/26/2016. Protein-protein interactions were downloaded from the *Homo sapiens* STRING v.10.0 network [16] using the aggregate score of all evidence types and were considered as interactions if they had ‘medium confidence’ or higher (interaction score ≥ 0.4). Population germline minor allele frequencies were derived from the phase three release of the 1000 Genomes Project [17].

### Identification of germline-somatic variant co-occurrences

For each patient, we compared their somatic and germline variant call files and flagged somatic variants that were within two nucleotides of a germline variant and predicted to affect the same amino acid as the germline variant. To visualize whether the variants were in *cis* or *trans*, and for additional support that the germline variant was a true call, we examined the aligned reads from the tumor sample in the region of each candidate interaction using IGV [18] and igv_plotter. Only co-occurrence events in *cis* and supported by multiple reads were considered to be germline-somatic variant events for subsequent analysis.

### Predicting SNV impact and germline-somatic co-occurrence impact

Variant impact was predicted using the Evolutionary Action (EA) method [19], which won multiple CAGI challenges in 2015, 2013, and 2011 [20]. In summary, the actions (EAs) of missense SNVs were calculated as the product of the evolutionary gradient *∂f/∂ri* and the perturbation magnitude of the substitution, Δ*ri*,*X*→*Y*. The evolutionary gradient was measured by importance ranks of the Evolutionary Trace method [21–23], and the perturbation magnitude by amino acid substitution odds. This approach produced scores on a continuous scale between 0 and 100, where a higher value indicated a larger predicted impact on protein fitness resulting from the amino acid substitution. Evolutionary action calculations are described at greater length in the original publication of the method [19]. Synonymous variants were given a heuristic score of zero, while stopgain variants were given a heuristic score of one hundred. When a variant affected multiple isoforms of a protein, the impact score was averaged across all affected isoforms. Using the maximum rather than average impact score for variants that affected multiple isoforms produced highly similar results, and both maximum and average impact values are included in the supplementary information (S1 Table).

## Results

### Detection of germline-somatic variant co-occurrence events

**Fig 2:**
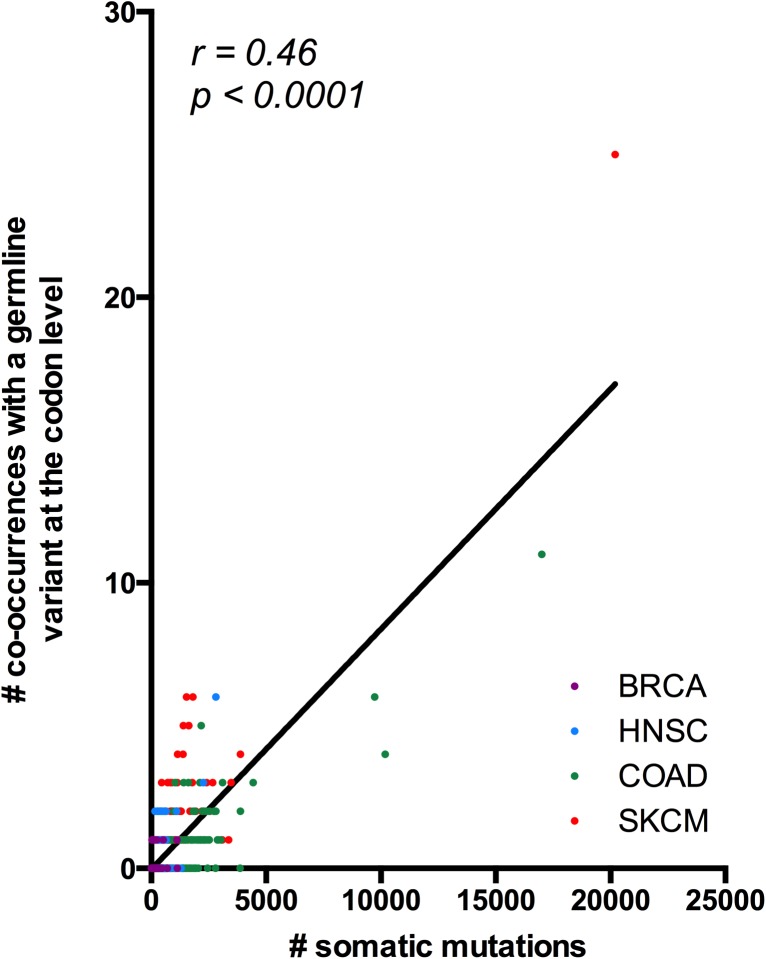
Linear relationship between somatic variant burden and codon-level co-occurrences between germline and somatic variants. Points denote individual patients and are colored by tumor type; solid line represents the linear regression on all points. The number of co-occurrences was calculated as the number of affected codons per patient, regardless of whether a codon was affected at two or three total positions.

In order to assess the prevalence of codon-level co-occurrences between germline and somatic variants, we compared the germline and somatic variant calls of four tumor types: head and neck squamous cell carcinoma (HNSC), skin cancer (SKCM), colon adenocarcinoma (COAD), and breast cancer (BRCA) to identify pairs of variants that may affect the same codon. These candidate events were then visualized in the tumor read alignment to establish that they affected the same allele. Across 1395 patients, we detected 604 candidate codon-level co-occurrences between germline and somatic variants, and confirmed 392 of these cases to be in *cis* (S1 Table). The vast majority of cases were composed of one germline and one somatic variant, and demonstrated strong bias toward pairs that had at least one variant affecting the third position of the codon (p < 0.001; chi square test), as would be predicted given that a disproportionate fraction of germline SNPs occur in the third codon position [12]. After accounting for pre-existing germline bias for the third codon position, there appeared to be no selection for any pair configurations (p = 0.35; chi square test) (S1 Fig). Six events resulted in changes to all three bases in the codon; in four cases two somatic variants and one germline variant were involved, while in two cases one somatic variant and two germline variants were involved. Overall, 17% of patients had at least one confirmed co-occurrence event, but the percentage of the cohort affected varied by tumor type, from 2.8% in BRCA to 34.7% in SKCM, reflecting the different somatic mutation rates of the cancer types examined. We found a strong proportional relationship between the number of somatic variants called in the patient and the number of co-occurrence events with the germline; this correlation was significant both when the patients were considered collectively (Spearman r = 0.46, p < 0.0001), as well as when they were separated by cancer type (p < 0.0001 for HNSC, COAD, SKCM, p = 0.018 for BRCA) (Fig 2). While many patients exhibited at least one such event, we found that only a very small fraction (0.07%) of all somatic variants co-occurred with germline variants at the codon level. These data show that codon-level co-occurrences are detectable but rare.

### Characterization of germline-somatic variant co-occurrence events

**Fig 3:**
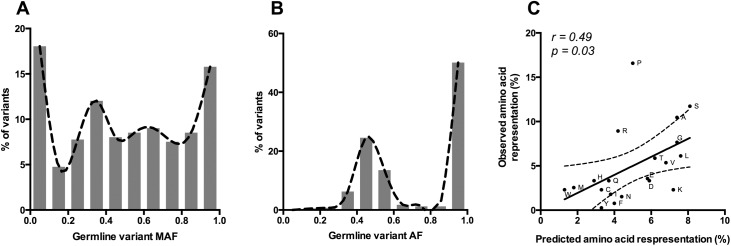
Characterization of germline-somatic variant co-occurrences at the codon level. (A) MAFs of germline variants involved in co-occurrence events. MAFs were derived from all individuals included in the Thousand Genomes Project Phase 3 release. (B) Allelic fraction of germline variants involved in co-occurrence events. (C) Correlation between predicted and observed representation of affected amino acids in co-occurrence events. Each point represents an amino acid labeled with its one-letter identifier. Solid line represents the linear regression and dotted lines represent the 95% confidence interval.

When somatic variants affected the same codon as a germline variant, the germline variants involved were found to be common in the human population. Of these variants, 92.2% had been annotated in phase three of the 1000 Genomes Project, and the average minor allele frequency (MAF) of these variants across all individuals was 0.49 (Fig 3A), more than thirty times higher than the average MAF for unique variants represented in the 1000 Genomes Project (p <0.001), but very similar to the average MAF of 0.50 for all variants represented in the 1000 Genomes project (z score = 0.56) (S2 Fig). There was no significant difference between the minor allele frequencies of these variants when calculated using all individuals or using only the population most representative of patient ethnicity (p = 0.1). These results indicate that co-occurrences primarily involve common SNPs, and that the MAF distribution and preference for common SNPs that we observe in the co-occurrence events reflect what would be expected from a random process. The germline variants involved in co-occurrence events were nearly evenly split between homozygous (53%), and heterozygous (47%) genotypes (Fig 3B). All possible underlying amino acids were affected by germline-somatic variant co-occurrences, and the representation of each amino acid in our data was correlated to the relative representation of the amino acids in vertebrate proteins overall (p = 0.03, r = 0.49) (Fig 3C) [24]. Prolines were, nonetheless, highly overrepresented (p<0.001; 1-sample proportion test). Rather than indicating meaningful positive selection for events in prolines, this outlier is likely due to the biased somatic mutation spectrums of the skin cancer and head and neck cancer cohorts; all proline codons are composed of at least two cytosines and the spectra of these cancers disproportionately target cytosine bases. These data show that codon-level co-occurrences primarily affect codons harboring common germline SNPs, and occur as a random process.

### Predicted impact of germline-somatic variant co-occurrence events on protein fitness

**Fig 4:**
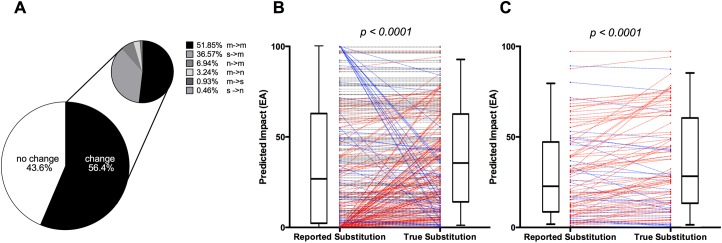
Impact of codon-level germline-somatic variant co-occurrences on prediction accuracy. (A) Representation of different types of incorrect variant predictions. If the amino acid substitution given by somatic calling was not changed by considering the germline context, the interaction was considered to be ‘no change’. If there was a change, data was split by whether it changed to a nonsense (*n*), silent (*s*), or different missense (*m*) substitution. (B) Paired EA impact of all co-occurrence events. Blue lines indicate pairs in which the reported substitution overestimated impact, red lines indicate pairs which the reported substitution underestimated impact, and grey indicates no change. Adjacent box plots show distributions of overall groups. (C) Paired EA impact of all co-occurrence events in which one missense substitution was incorrectly called as another. Blue lines indicate pairs in which the reported substitution overestimated impact and red lines indicate pairs which the reported substitution underestimated impact. Adjacent box plots show distributions of overall groups.

In order to assess how codon-level co-occurrences between somatic and germline variants affect the reliability of somatic variant reporting, we next quantified how often these events led to a different predicted substitution in the tumor. We found that the true amino acid substitution in the tumor was different from the reported annotation the majority (56.4%) of the time (Fig 4A). When there was annotation error, in about half of cases the true substitution was a different missense variant from the one reported; however, in about 4% of cases the true change was a stopgain rather than a silent or missense substitution, and in 37% of cases the true change was a missense rather than a silent substitution. For these events, the true substitution in the tumor was much less conservative than a traditional somatic variant calling schema would report.

To test whether the true substitutions were biased toward being more impactful than those reported, we compared the Evolutionary Action (EA) scores produced when the germline context was considered during variant calling to those produced when it was not. We found that across the entire set of variants, EA scores were significantly higher when the germline context was considered (p < 0.0001; Wilcoxon matched-pairs signed ranks test) (Fig 4B). When we focused on the set of variants in which the annotation difference was between two missense variants, the predicted impact was still significantly higher when the germline context was considered (p < 0.0001; Wilcoxon matched-pairs signed ranks test), with an average EA score increase of 51% (Fig 4C). These data show that codon-level co-occurrence events do usually cause the annotation of the amino acid substitution to be incorrect, and its impact on protein fitness to be underestimated even in cases where the class of mutation remains the same.

### Germline-somatic variant co-occurrences as potential cancer driver events

**Fig 5:**
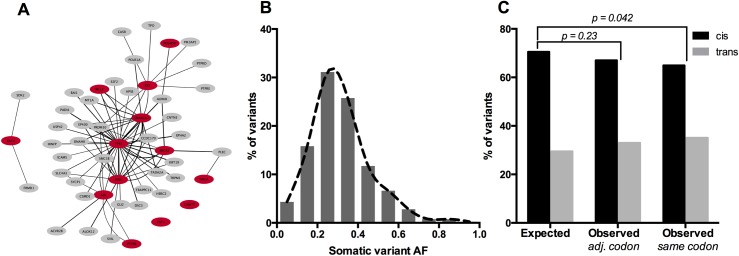
Cancer genes are affected by co-occurrence events but co-occurrence events do not exhibit positive selection signals. (A) COSMIC cancer genes affected by co-occurrence events and their affected network partners. Interactions were defined as having medium confidence or higher in STRING v.10.0, and were visualized in Cytoscape. (B) Variant allele fractions of all somatic variants involved in co-occurrence events with the germline. (C) Ratio of somatic variants found to be *in cis* versus *in trans* with germline variants. The expected ratio was derived from the allelic fractions of all germline variants in each cohort. Observed ratios were calculated for somatic variants that were candidates for affecting the same codon as a germline variant (*same codon*), and for somatic variants that were 1–2 nts away from a germline variant but predicted to affect a different codon (*adj*. *codon*).

To assess if the detected codon-level co-occurrence events could result in driver mutations of cancer, and to determine whether these events undergo positive selection, we first identified affected genes considered to be cancer-promoting by the COSMIC cancer gene census. In our data, 13 COSMIC cancer genes were impacted by a total of 14 co-occurrence events (Table 1). We found that exactly half of these co-occurrences resulted in a disparity between the reported and true amino acid substitution, which is not significantly different than the percentage seen for non-cancer genes. Interactions that did not change the substitution occurred in TP53, RET, SMARCA4, ESR1, BRCA2, CNOT3 and FAT4, while interactions that did change the substitution were found in ALK, PDE4DIP, NACA, PTPRB, FAT1, and MLL3. MLL3 exhibited germline-somatic co-occurrences in two different skin cancer patients, and in both cases produced a substantial increase in the predicted impact of the variant when the germline context was considered. However, although the average change in predicted EA was slightly higher overall for co-occurrences that involved COSMIC genes, this increase was not significant (p = 0.36; unpaired t-test). In addition, although there were a number of events in COSMIC genes, as well as over three dozen events in protein network interactors of the affected COSMIC genes (Fig 5A), there was no detectable enrichment for COSMIC cancer genes amongst all affected genes (p = 0.29; hypergeometric distribution test). These data show that while cancer-related genes are in some cases affected by codon-level co-occurrences of somatic and germline variants, there does not appear to be any positive selection for events occurring in cancer genes, and events occurring in these genes do not exhibit different properties or significantly larger impacts when compared to non-cancer genes.

**Table 1:** COSMIC cancer genes affected by co-occurrence events.

Cancer Type	Gene	Impact	Reported Substitution (somatic only)	True Substitution (germline+somatic)^a^	Change in impact score (ΔEA)^b^
**HNSC**	TP53	*no change*	R119H	R119H	0.0
**HNSC**	SMARCA4	*no change*	A503E	A503E	0.0
**COAD**	ALK	*silent->miss*	I1461I	I1461V	0.1
**COAD**	RET	*no change*	A432V	A432V	0.0
**COAD**	PDE4DIP	*miss->miss*	C871Y	C871H	-3.0
**SKCM**	NACA	*silent->miss*	V226V	V336D	40.4
**SKCM**	ESR1	*no change*	R243C	R243C	0.0
**SKCM**	PTPRB	*miss->miss*	R94S	R94N	1.4
**SKCM**	MLL3	*miss->miss*	T316I	T316F	15.0
**SKCM**	CNOT3	*no change*	P243L	P243L	0.0
**SKCM**	FAT4	*no change*	R4866K	R4866K	0.0
**SKCM**	MLL3	*silent->miss*	T316T	T316S	34.0
**SKCM**	FAT1	*silent->miss*	V482V	V482I	39.6
**SKCM**	BRCA2	*no change*	L1521L	L1521L	0.0

HNSC, head and neck squamous cell carcinoma; COAD, colon adenocarcinoma; SKCM, skin cutaneous melanoma.

^a^True substitution indicates the substitution that is present in the tumor tissue and considers both germline and somatic variant contributions.

^b^ΔEA was calculated by subtracting the EA score of the substitution produced when considering only the somatic variant from the EA score of the substitution produced when considering both the somatic and germline variants within the codon.

Since not all potential cancer driver genes have been identified and catalogued, we also assessed the behavior of the somatic variants in the tumor samples. Since variants that drive tumor cell growth should be present in a larger fraction of the tumor, we quantified the tumor variant allele fractions for all 392 somatic variants involved in co-occurrence events with germline variants. These somatic variants demonstrated a single allelic peak centered around a variant allele fraction of 0.3, reflecting the clonal heterogeneity of tumor composition (Fig 5B). Overall, the average variant allele fraction (VAF) of somatic variants that co-occurred in codons with germline variants was not was not significantly higher than the average VAF of those that did not (p = 0.056; two-sided independent t test) (S3 Fig). When we restricted the analysis to somatic variants that not only interacted with germline variants but also resulted in a change to the reported substitution, the average VAF was also not significantly higher than expected (p = 0.34; two-sided independent t test). These data indicate that, at least when considered collectively, amino acid substitutions resulting from germline-somatic co-occurrences at the codon level are unlikely to act as drivers of tumorigenesis.

As another measure of selection, we considered the ratio of the original 604 candidate variants that were assessed for whether they occurred *in cis* or *in trans* with a germline variant. Using the distributions of homozygous and heterozygous genotypes for all germline variants in each cohort, we calculated the expected ratios of *cis* and *trans* events given that a homozygous germline variant would always result in a co-occurrence while a heterozygous variant would be *in cis* with a somatic variant only half the time. We found that across all patients, candidate germline-somatic co-occurrence events were depleted for *in cis* events, with the germline and somatic variants affecting different alleles more often than would be expected by chance given the composition of the patient exomes (p = 0.042; 2-sided Fisher’s exact test). When we instead compared the expected ratio to the *cis* vs *trans* ratio of somatic variants that occurred at the same (1–2 nt) distance to a germline variant but did not affect the same codon, we found that there was no significant deviation from the expected distribution given by the patient exomes (p = 0.23; 2-sided Fisher’s exact test) (Fig 5C). These data indicate that rather than exhibiting signs of positive selection, germline-somatic co-occurrences may actually undergo negative selection.

## Discussion

We detected hundreds of codon-level co-occurrences of germline and somatic variants, including over a dozen in well-known cancer genes. However, the germline-somatic co-occurrence events we detected do not appear to be a source of driver events promoting tumorigenesis. Amongst candidate events there is no positive selection for the germline and somatic variant occurring *in cis*, and when co-occurrences do occur *in cis* they do not show preference for cancer genes and are not associated with a higher allele fraction in the tumor. Although pre-existing germline variants can affect cancer development and interact indirectly with tumor-specific variants by influencing which have greatest benefit to the tumor and undergo positive selection, positive selection for germline-somatic interaction does not appear to occur at the codon level. Instead, these events appear to occur either as a random process or one under mild negative selection; co-occurrences exhibit no preference for particular codon positions or underlying amino acids, and the number of codon-level co-occurrences in a patient shows a direct proportional relationship with the number of somatic mutations in the tumor. The fact that we see significantly fewer than expected *in cis* pairs of germline and somatic variants when they act on the same codon, but not when they are at the same distance apart but acting on different codons, provides support for the possibility that germline-somatic co-occurrences within the same codon could actually be harmful to the tumor and be selected against.

Regardless of their contribution to cancer development, co-occurrence events that do occur are unlikely to be detected by current methods for somatic mutation calling, which treat the germline sample as a reference from which to subtract candidate somatic variants, rather than as a source of potential variant collaboration. We found that these events led to imprecise annotation of somatic variants in the majority of cases. When the germline contexts of the somatic variants were considered, the amino acid changes and their predicted impacts were significantly larger, even for annotations that did not change the mutation type. Traditional somatic calling, therefore, underestimates the impact of the substitution actually present in the tumor cells. However, these inaccuracies are unlikely to cause problems for large-scale mutation-based analyses of cancer because they comprise a small percentage of mutations overall and occur primarily in genes unaffiliated with cancer.

We conclude that these events often lead to imprecise annotation of somatic variants but do not appear to be a source of driver events during cancer development. While we do not find evidence for cancer driver events caused by germline and somatic variant interaction at the codon level, the relatively small sample size of co-occurrences taking place in cancer genes limits our ability to close the door entirely on the possibility that some of these events may produce drivers. Moreover, interactions may yet be under selection at the single-gene level, perhaps interacting within an allele but from a larger distance. Multiple variants within the same gene can have additive or non-additive effects on protein fitness, and the impact of these events on tumorigenesis has not been fully explored. Additional work is necessary to determine whether germline and somatic variant interactions undergo positive selection on a level higher than the codon and lower than the multi-gene scale.

## Supporting information

S1 Fig Distribution of codon position pairs affected by co-occurrences between germline and somatic variants. Expected percentages are based on a model that reflects the proportion of germline variants in each codon position across all patients and assumes somatic variants have no codon preference.
(TIFF)

S2 Fig MAF distribution for germline variants that co-occur with somatic variants. (A) Observed MAF distribution for germline variants that co-occur with somatic variants (red) compared to the MAF distribution for all 1000 Genomes Project variants (black). (B) Distribution of simulated average MAF values. 1000 sets of 392 variants were drawn randomly from the complete set of 1000 Genomes Project variants; for each draw, the MAF was averaged. The observed average MAF of germline variants involved in codon-level co-occurrences with somatic variants is denoted in red.
(TIFF)

S3 Fig Somatic variant allele fraction distributions. Variant allelic fraction distribution for somatic variants that co-occur with germline variants (red), for somatic variants in COSMIC cancer genes that co-occur with germline variants (orange), for somatic variants that co-occur with germline variants and affect the reported substitution (blue), and for all somatic variants in the cohorts studied (green).
(TIFF)

S1 Table Codon-level co-occurrences between germline and somatic variants. (XLSX)
