# Supplementary figures and images for: Codon-level co-occurrences of germline variants and somatic mutations in cancer are rare but often lead to incorrect variant annotation and underestimated impact prediction
Source: PLoS One. 2017 Mar 28;12(3):e0174766. doi: 10.1371/journal.pone.0174766 (PMC5370158; doi:10.1371/journal.pone.0174766)

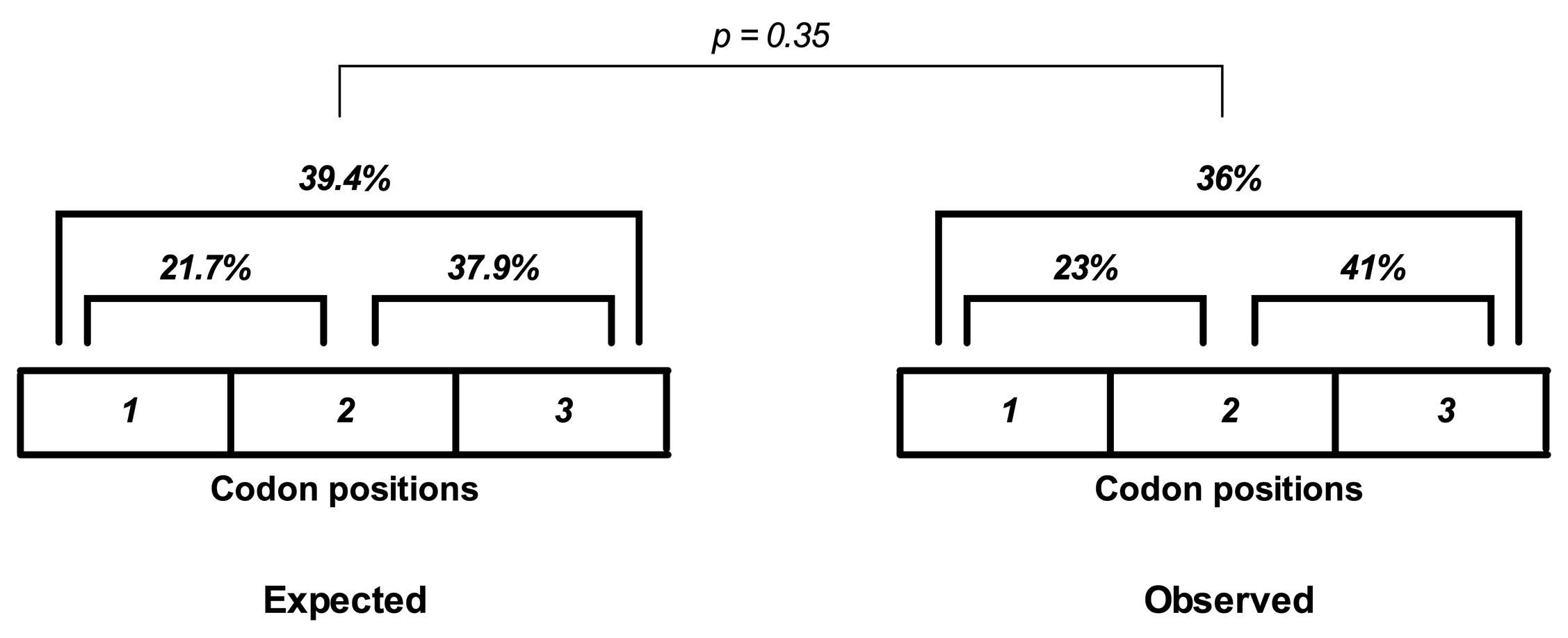

Supplement: S1 Fig — Expected percentages are based on a model that reflects the proportion of germline variants in each codon position across all patients and assumes somatic variants have no codon preference. (TIFF) [file pone.0174766.s001.tiff]
